# SRA Regulates Adipogenesis by Modulating p38/JNK Phosphorylation and Stimulating Insulin Receptor Gene Expression and Downstream Signaling

**DOI:** 10.1371/journal.pone.0095416

**Published:** 2014-04-17

**Authors:** Shannon Liu, Ruichuan Xu, Isabelle Gerin, William P. Cawthorn, Ormond A. MacDougald, Xiao-Wei Chen, Alan R. Saltiel, Ronald J. Koenig, Bin Xu

**Affiliations:** 1 Department of Internal Medicine, Division of Metabolism, Endocrinology and Diabetes, University of Michigan Medical Center, Ann Arbor, Michigan, United States of America; 2 Department of Molecular & Integrative Physiology, University of Michigan Medical Center, Ann Arbor, Michigan, United States of America; 3 Life Science Institute, University of Michigan, Ann Arbor, Michigan, United States of America; 4 Department of Internal Medicine, University of Michigan Medical Center, Ann Arbor, Michigan, United States of America; Florida International University, United States of America

## Abstract

The Steroid Receptor RNA Activator (SRA) enhances adipogenesis and increases both glucose uptake and phosphorylation of Akt and FOXO1 in response to insulin. To assess the mechanism, we differentiated ST2 mesenchymal precursor cells that did or did not overexpress SRA into adipocytes using combinations of methylisobutylxanthine, dexamethasone and insulin. These studies showed that SRA overexpression promotes full adipogenesis in part by stimulation of insulin/insulin-like growth factor-1 (IGF-1) signaling. SRA overexpression inhibited phosphorylation of p38 mitogen activated protein kinase (MAPK) and c-Jun NH2-terminal kinase (JNK) in the early differentiation of ST2 cells. Conversely, knockdown of endogenous SRA in 3T3-L1 cells increased phosphorylation of JNK. Knockdown of SRA in mature 3T3-L1 adipocytes reduced insulin receptor (IR) mRNA and protein levels, which led to decreased autophosphorylation of IRβ and decreased phosphorylation of insulin receptor substrate-1 (IRS-1) and Akt. This likely reflects a stimulatory role of SRA on IR transcription, as transfection studies showed that SRA increased expression of an IR promoter-luciferase reporter construct.

## Introduction

Obesity is closely associated with a number of diseases including type 2 diabetes, cardiovascular disease, hypertension, cancer and gallstones. Adipocytes function both as reservoirs of fuel and as endocrine cells, secreting adipokines such as leptin, adiponectin, interleukin-6 and tumor necrosis factor-a to regulate whole-body energy metabolism and glucose homeostasis [1], [2]. Adipogenesis is a complex process that is highly regulated by coordinated effects of numerous transcription factors and signaling molecules, including peroxisome proliferator-activated receptor gamma (PPARγ) [3], [4], the CCAAT/enhancer-binding proteins (C/EBPs) [5], [6], Kruppel-like factors (KLFs) [7], Wingless proteins (Wnts) [8], and E2Fs [9].

Both 3T3-L1 preadipocytes and bone marrow-derived ST2 adipocyte precursors can be differentiated in cell culture into mature adipocytes by standard hormone cocktails that include fetal bovine serum (FBS), 3-isobutyl-1-methylxanthine (IBMX), dexamethasone (Dex) and insulin [10], [11]. IBMX and Dex are important for preadipocyte differentiation, whereas insulin plays unique and important roles in both adipocyte differentiation and mature adipocyte function. Insulin is postulated to regulate adipogenesis by activating extracellular signal-regulated kinase (ERK) and p38 kinase [5], [12], and/or critical signaling components such as insulin receptor substrate-1 (IRS-1) [13], [14], Akt [15], [16] and mTOR [17]. However, the molecular mechanisms through which insulin promotes adipogenesis are not fully understood.

After terminal differentiation, adipocytes in culture increase lipogenesis and gain sensitivity to insulin through expression of proteins such as PPARγ, C/EBPα, adiponectin, Glut4, insulin receptor (IR) and IRS-1. Insulin stimulates glucose uptake, utilization and storage through binding to the IR, which triggers autophosphorylation of the IR β-subunit [18], activation of IRS-1 by tyrosine phosphorylation, and activation of downstream signaling through the phosphatidylinositol 3-kinase (PI3K)-Akt/protein kinase B, Ras-mitogen-activated protein kinase (MAPK), and Cbl-CAP pathways [18], [19], [20]. Given the central role of the IR, it is important to note that the hyperinsulinemia accompanying insulin-resistant states such as obesity and type 2 diabetes can be associated with lowered IR levels [21], [22], [23].

The *Sra1* gene expresses a steroid receptor RNA activator (SRA) that was initially found to be a transcriptional coactivator for steroid receptors [24]. It has subsequently been found to serve as a coactivator for numerous transcription factors [25], [26], [27], [28], but the biological functions of SRA are largely unknown. We have recently shown that SRA functions as a coactivator of PPARγ and promotes adipocyte differentiation [29]. Our gene profiling experiments revealed hundreds of SRA-responsive genes in adipocytes, but the molecular mechanisms by which SRA enhances adipogenesis and insulin-stimulated glucose uptake remain to be elucidated. By alternative splicing, *Sra1* also encodes an SRA protein (SRAP) [30], [31], although the function of SRAP is largely unknown. In this study, we report that SRA regulates signaling events early in preadipocyte differentiation. In mature adipocytes SRA increases insulin receptor (IR) transcription and IR protein content, which results in increased insulin-responsive phosphorylation of the IR and downstream targets such as IRS-1 and Akt.

## Materials and Methods

### Cell Culture, Staining and Reagents

Mouse 3T3-L1 preadipocytes and human embryonic kidney 293T cells were obtained from the American Type Culture Collection (ATCC) and maintained in Dulbecco’s modified Eagle’s medium supplemented with 10% calf serum and penicillin-streptomycin at 37°C in 10% CO_2_. Mouse marrow-derived ST2 cells were obtained from the Riken Bioresource Center-Cell Bank and incubated at 37°C in 5% CO_2_ in α-minimal essential medium supplemented with 10% FBS and penicillin-streptomycin. Induction of 3T3-L1 or ST2 cell differentiation was performed as described [29]. Briefly, 2 day post-confluent cells (day 0) were fed with media supplemented with 10% FBS and a hormone cocktail containing IBMX (0.5 mM), dexamethasone (1 µM) and insulin (0.167 mM), denoted MDI. On day 2, the cells were treated again with 0.167 mM insulin, and subsequently were refed with growth media containing 10% FBS every 2 days. In some studies, troglitazone (50 mM in dimethylsulfoxide) was added to the hormone cocktail to achieve a final media concentration of 5 µM (MDIT). Lipid accumulation in adipocytes was visualized by micrographs or staining with Oil Red O as described previously [29].

Antibodies against the following proteins were obtained as indicated: SRAP (Cat# A310-226A, Bethyl Laboratories, Montgomery, TX); Phospho-p38 MAPK (Thr180/Tyr182) (3D7) (Cat# 9215), Phospho-p44/42 MAPK (thr202/Tyr204) (D13.14.4E) (Cat# 4370), p38 MAPK (Cat# 9212), p44/42 MAPK (137F5) (Cat# 4695), Insulin Receptor β (4B8) (Cat# 3025), IRS-1 (Cat# 2382), phospho-SAPK/JNK (Thr183/Tyr185) (81E11) (Cat# #4668), SAPK/JNK (56G8) (Cat# 9258), JNK1 (2C6) (Cat# 3708), JNK2 (Cat# 4672), JNK3 (55A8) (Cat# 2305), β-actin (Cat# 4967), Phospho-Insulin Receptor β (PY1345) (Cat# 3026), Phospho-Insulin Receptor β (PY1361) (Cat# 3023), Phospho-Akt (Thr308) (Cat# 9275) and Akt (Cat# 9272) from Cell Signaling Technology (Danvers, MA); Phospho-Insulin Receptor β (pY972), Phospho-IRS-1 (pY941) and Phospho-IRS-1 (pY612) from Invitrogen (Carlsbad, CA); Phospho-Insulin Receptor β (pY1328) from BioSource (Camarillo, CA); and phosphotyrosine (pY4G10) from Millipore (Cat# 05-321) (Billerica, MA).

### Gene Silencing by Short Hairpin RNA (shRNA)

A 21-nucleotide shRNA construct targeting mouse SRA1 mRNA was cloned into the retroviral pSUPERIOR.retro.puro vector (OligoEngine (Seattle, WA)) or the pLentiLox3.7-GFP vector with a sense-loop-antisense design, using the loop sequence CTTCCTGTCA as described [29].

### Plasmids, Transfection and Retroviral Infection

The human SRA isoform 2 expression vector pSCT-SRA (non-protein coding) was kindly provided by Dr. Rainer Lanz (Baylor College of Medicine, Houston, TX) [24]. The pMSCV retroviral expression vector and pMSCV-SRA were described previously [29], [32]. Retroviral transduction of ST2 or 3T3-L1 cells for stable overexpression of pMSCV/pMSCV-SRA or knockdown of endogenous SRA by either retroviral or lentiviral transduction of shRNA against SRA and shControl was performed as described previously [29]. The plasmid pSCT-SRA (denoted SRA Only), contains the human SRA RNA core sequence and hence expresses SRA but not SRAP. By alternative splicing the *SRA1* gene also can encode a protein, SRAP [31], [33]. Human full-length SRAP cDNA (hSRAP) was amplified by PCR using cDNA template that was reverse transcribed from total RNA of HepG2 cells. The amplified hSRAP cDNA with Sal1 and Kpn1 overhangs was ligated into the pSCT vector to derive pSCT-hSRAP (denoted SRA-WT). pSCT-hSRAP expresses both the full length SRA RNA and SRAP. SRAP mutations including a point mutation and a series of silent mutations in SRA RNA stem loops 1 and 7 were constructed by inverse PCR and are described later. The introduction of silent nucleotide mutations in the SRA stem loops 1 and 7 which disrupted the RNA stem loop structure and impaired its coactivation was described previously [34]. pGluc-Basic containing a reporter gene but lacking promoter elements was obtained from the New England BioLabs. pIRP-GLuc, in which the insulin receptor promoter (−1718 to +106 bp relative to the most 5′ transcription start site) drives expression of Gaussia Luciferase, was a gift from Drs. R. Singh and A. Mani (Yale University School of Medicine, New Haven, CT) [35]. Transient transfections were performed as described previously [27].

### Cell Lysis and Immunoblotting

Cells were lysed in buffer containing 40 mM HEPES, 120 mM sodium chloride, 10 mM sodium pyrophosphate, 10 mM sodium glycerophosphate, 1 mM EDTA, 50 mM sodium fluoride, 0.5 mM sodium orthovanadate and 1% Triton X-100. Cell lysates were gently resuspended and incubated at 4°C with gentle rocking for 40 min to 1 h, followed by microcentrifugation for 10 min at 4°C. The supernatants were transferred to new tubes and protein concentrations were determined. Proteins were separated by SDS-PAGE and transferred onto polyvinylidene difluoride membranes, and immunoblotting was performed using the antibodies described above. Detection by enhanced chemiluminescence was with a SuperSignal West Dura kit (Thermo Fisher Scientific, Rockford, IL) and a Bio-Rad Fluor-S Max Multi-Imager.

### Gene Expression Analysis

For reverse transcription-real time quantitative PCR (RT-qPCR) analysis of mRNA expression, total RNA was first isolated from cells using Trizol reagent. Reverse transcription of RNA to cDNA and analysis of relative mRNA levels by RT-qPCR were done as described [27], [29]. Sequences of the qPCR primers are available upon request or have been previously described [29].

### Luciferase Reporter Gene Assay

3T3-L1 preadipocytes were cotransfected with pIRP-GLuc or pGluc-Basic (100 ng) and either pSCT or pSCT-SRA, pSCT-SRAP or pSCT-SRAP silent mutant plasmids for 48 hr using Lipofectamine Plus Reagent (Invitrogen) in 24 well plates, and luciferase activity was measured using a BioLux Gaussia Luciferase Assay Kit (New England Biolabs, Inc.).

### Statistical Analysis

Results are presented as the mean±SD. When comparing two groups, significance was determined using Student’s *t* test. When more than two groups were compared, an analysis of variance (ANOVA) was followed by Scheffe’s test, and the significance is indicated as *p<0.05; **p<0.01, and ***p<0.001.

## Results

### SRA Overexpression Enhances Adipogenesis in ST2 Adipocyte Precursors in an Insulin-dependent Manner

**Figure 1:**
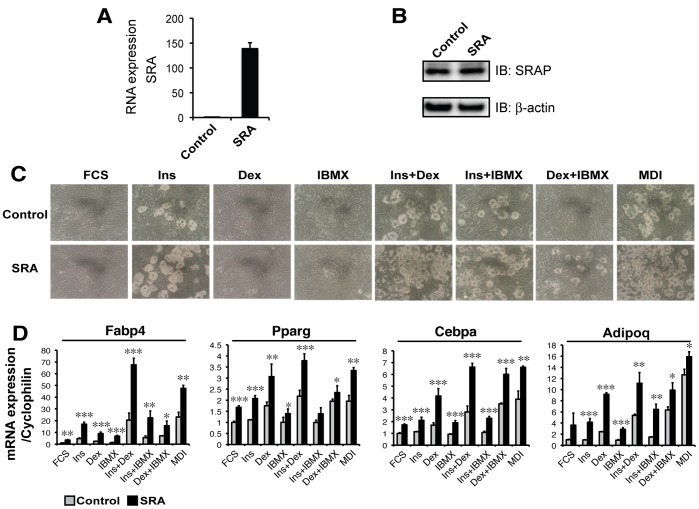
SRA overexpression enhances adipogenesis in ST2 adipocyte precursors in an insulin-dependent manner. ST2 cells were retrovirally transduced with an SRA expression vector (pMSCV-SRA, denoted as SRA) or empty vector control (pMSCV, denoted as Control). A, Stable overexpression of SRA were confirmed by RT-qPCR using human SRA primers. Transcript expression was normalized to Ppia (cyclophilin A) and is presented as mean ± S.D. relative to the SRA expression determined in control cells set at 1. B, Immunoblot using an SRAP specific antibody indicated similar endogenous SRA protein (SRAP) expression in Control and SRA overexpressing ST2 cells. The same membrane was re-probed with anti-β-actin as a loading control. C, Cells were induced to differentiate into adipocytes by treatment with fetal calf serum (FCS), insulin (Ins), dexamethasone (Dex), methylisobutylxanthine (IBMX), or the indicated combinations. Micrographs of cells at day 4 post-induction indicate lipid accumulation. D, Expression of Fabp4, Pparg, Cebpa, or Adipoq (adiponectin) was determined by RT-qPCR at the end of differentiation at day 4. Transcript expression was normalized to Ppia (cyclophilin A) and is presented as mean ± S.D. relative to expression in FCS-induced control cells set at 1. Statistical significance was evaluated with Student’s t test: *p<0.05, **p<0.01 and ***p<0.001. Results are representative of three independent experiments.

Our previous studies showed that overexpression of SRA enhances adipogenesis in ST2 precursors induced with full hormonal cocktail (MDI) but has little effect on spontaneous adipogenesis in the absence of MDI [29]. Therefore, we hypothesized that SRA influences adipogenesis by regulating pathways targeted by MDI. To address this hypothesis, ST2 adipocyte precursors stably containing either an empty control vector pMSCV (Control) or pMSCV-SRA (SRA) were established, in which SRA RNA was overexpressed ∼140 fold (Figure 1A) but without SRAP overexpression (Figure 1B). ST2 Control or ST2 SRA overexpressing adipocyte precursors were induced to differentiate with full MDI or with single- or double-combinations of each component. Effects on adipogenesis were then evaluated at day 4 post-induction by assessing lipid droplet accumulation and expression of adipocyte marker genes. In agreement with a previous study [10], we found that lipid droplet formation was strongly induced with cocktails containing insulin (Figure 1C), although some lipid droplets also were apparent in SRA-expressing cells induced with Dex plus IBMX. Expression of the adipocyte marker Fabp4 (also known as aP2) also was most prominent in SRA over-expressing cells with cocktails containing insulin (Figure 1D), although the expression of other adipocyte markers (Pparg, Cebpa, Adipoq) was not as strictly dependent on insulin. These observations suggest that SRA enhances ST2 adipogenesis at least in part by sensitizing ST2 precursors to the pro-adipogenic effects of insulin.

**Figure 2:**
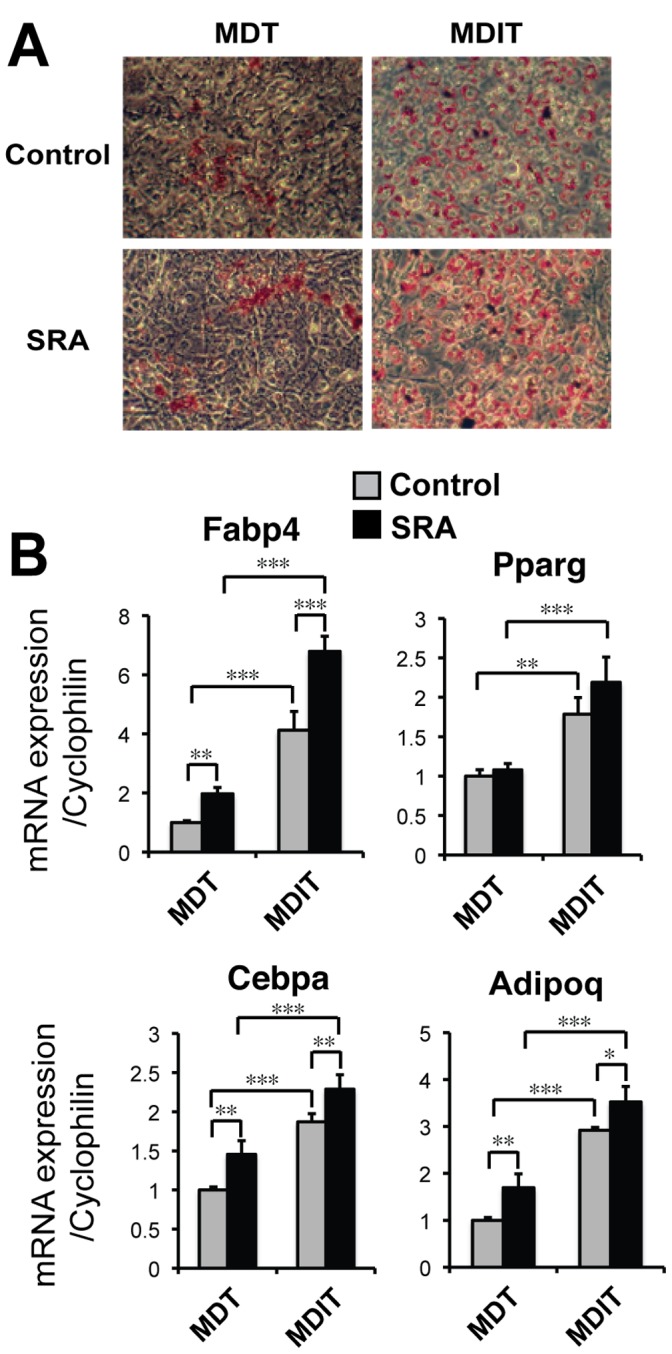
Differentiation of ST2 cells into adipocytes in the presence of a PPARγ agonist is insulin- and SRA-dependent. ST2 cells were transduced with either SRA expression vector (pMSCV-SRA) or empty control vector (pMSCV) and induced to differentiate by treatment with methylisobutylxanthine (M), dexamethasone (D) and troglitazone (T), without or with insulin (I). A, Oil Red O staining to identify triglyceride droplets. B, Expression of Fabp4, Pparg, Cebpa, and Adipoq (adiponectin) determined by RT-qPCR and normalized to Ppia (cyclophilin A). Results are mean ± S.D. relative to MDT-induced controls. Statistical significance was evaluated with Student’s *t* test, or ANOVA followed by Scheffe’s test: *p<0.05, **p<0.01 and ***p<0.001. Results are representative of three independent experiments.

Thiazolidinediones (TZDs) are adipogenic ligands for PPARγ, and as such TZDs induce many adipocyte PPARγ target genes [36], [37]. Given that SRA can function as an RNA coactivator for PPARγ [29], we asked whether insulin also is required for the pro-adipogenic effect of SRA even in the presence of a TZD (MDT vs. MDIT). Indeed, MDT without insulin produces very little lipid droplet accumulation, even with SRA overexpression (Figure 2A). At the gene expression level, MDIT induces the adipocyte markers Fabp4, Pparg, Cebpa and Adipoq more strongly than does MDT. However, the ability of SRA to induce these genes is similar with or without insulin, with the possible exception of Pparg (Figure 2B).

### SRA Regulates p38/JNK Activity during Early Preadipocyte Differentiation

**Figure 3:**
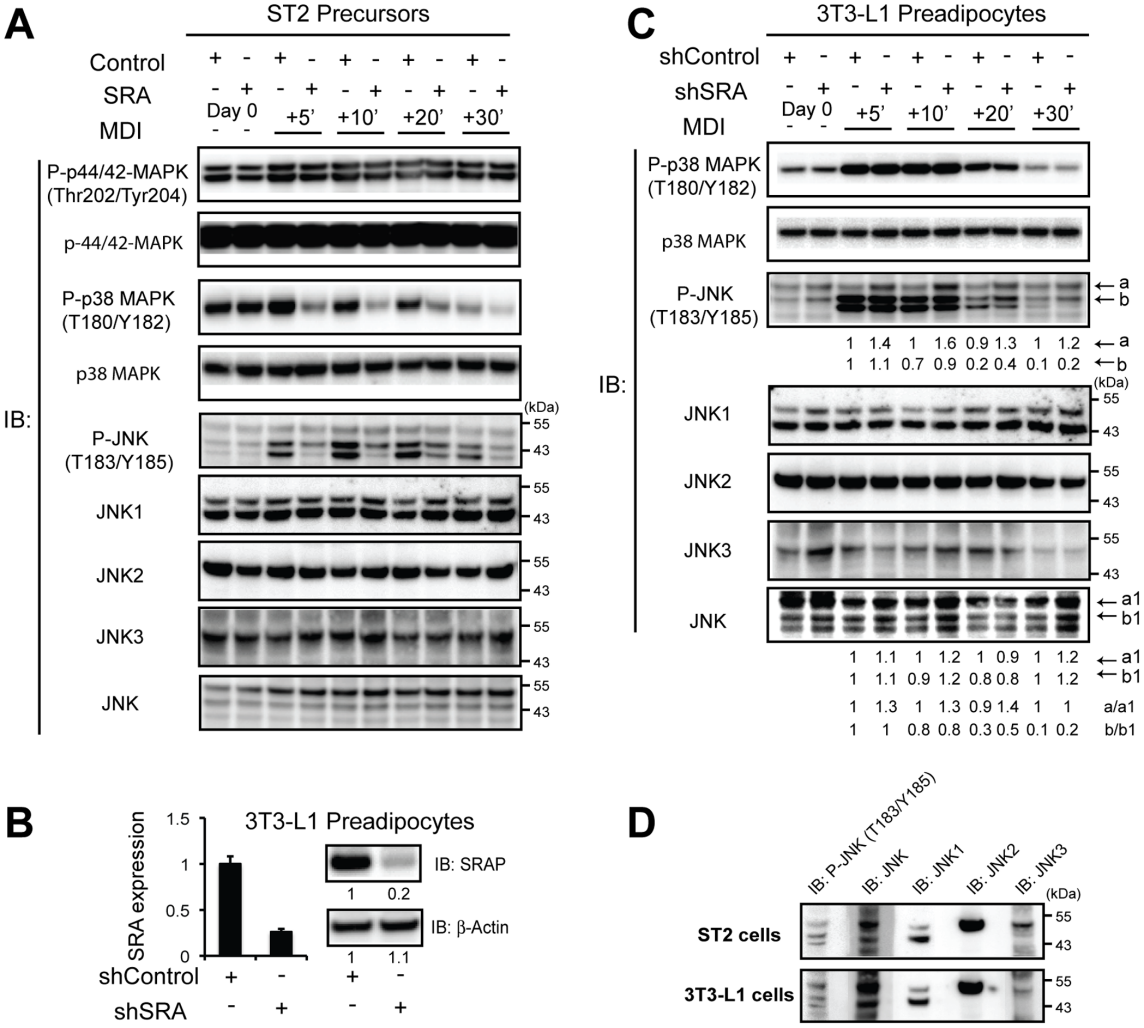
SRA regulates p38/JNK activity during early preadipocyte differentiation. A, Control or SRA-overexpressing ST2 cells were grown to confluence and induced to differentiate with MDI. Cell lysates were obtained at the indicated times post-MDI induction. Phosphorylation and total protein expression of p38, p44/42 and JNK was assessed by immunoblotting using specific antibodies, as indicated. B, 3T3-L1 preadipocytes with stable knockdown of SRA were generated by retroviral infection with an shRNA against SRA (shSRA); a scrambled shRNA was used to generate control preadipocytes (shControl). Left panel, stable knockdown of endogenous SRA RNA in 3T3-L1 preadipocytes was determined by RT-qPCR using mouse SRA primers. Transcript expression was normalized to Ppia (cyclophilin A) and is presented as mean ± S.D. relative to expression in shControl cells set at 1. Right panel, immunoblot using an SRAP specific antibody confirmed the effective knockdown of endogenous SRA protein (SRAP) in shSRA 3T3-L1 preadipocytes. Reprobing with anti-β-actin served as a loading control. Bands were quantified from immunoblot digital images using Bio-Rad Quantity One software, and the relative results are presented below each immunoblot image. C, shSRA and shControl 3T3-L1 preadipocytes were induced with MDI and protein phosphorylation assessed as described for cells in Figure 3A. Bands labeled a and b in the P-JNK immunoblot, and a1 and b1 in the total JNK immunoblot, correspond to the p54 and p46 kDa species and were quantified as stated above. Results in A, B and C are representative of three independent experiments. D, For ST2 cells, the sample +10 minutes MDI minus SRA was loaded into multiple lanes of one gel and immunoblotted. The membrane was cut so that each lane could be probed individually with an antibody to either phosphoJNK (P-JNK), total JNK (JNK), JNK1, JNK2 or JNK3. The lanes were reassembled to capture the digital image shown. A similar procedure was used for 3T3-L1 cells +10 minutes MDI +shSRA.

We next investigated how SRA expression affects signals downstream of insulin. Insulin influences adipocyte differentiation through regulation of MAPKs, including ERK1/2 (p44/p42), p38 and c-Jun amino-terminal kinase (JNK), each of which can regulate adipogenesis [12], . We previously showed that SRA is expressed at low levels in ST2 precursors and high levels in 3T3-L1 preadipocytes, and therefore we have used SRA overexpression in ST2 precursors and knockdown in 3T3-L1 preadipocytes to assess the potential roles of SRA in adipocyte biology [29]. We continued to use these two model systems in the present investigations. SRA overexpression in ST2 precursors (Figure 3A) or knockdown in 3T3-L1 preadipocytes (knockdown of both SRA and SRAP are confirmed in Figure 3B) did not affect phosphorylation of p44/42 (Figure 3A; data not shown). In contrast, stable SRA overexpression in ST2 precursors was associated with marked inhibition of p38 phosphorylation (Figure 3A). This suggests that SRA may enhance ST2 adipogenesis by suppressing p38 activation, because p38 activity likely inhibits adipogenesis [38]. However, stable knockdown of SRA did not affect p38 activation in 3T3-L1 preadipocytes (Figure 3C), even though adipogenesis is impaired in these cells [29].

In general, over-expression of SRA in ST2 cells inhibited, and knockdown of SRA in 3T3-L1 cells stimulated, JNK phosphorylation, although the specific details differed in the two cell lines (Figure 3). In ST2 cells, MDI increased the quantity of two low molecular weight (∼46 kDa) phosphoJNK (P-JNK) isoforms and SRA over-expression inhibited this phosphorylation (Figure 3A). A larger (∼54 kDa) P-JNK isoform was essentially unaffected by MDI and SRA over-expression. 3T3-L1 cells differed in that SRA knockdown did not affect phosphorylation of the smaller JNK isoforms, but it did induce phosphorylation of the larger JNK isoform (Figure 3C).

There are three genes, each with alternative transcripts, that encode JNK proteins, the end result of which is 10 JNK protein isoforms whose molecular weights are approximately 46 kDa (JNK1α1, JNK1β1, JNK2α1, JNK2β1 and JNK3α1) or 54 kDa (JNK1α2, JNK1β2, JNK2α2, JNK2β2 and JNK3α2) [42], [43]. Using antibodies specific for JNK1, JNK2 or JNK3, we found that all 3 are expressed in both ST2 and 3T3-L1 cells, although JNK3 has the weakest signal (Figures 3A and 3C). JNK1 shows bands representing both its p46 and p54 isoforms (α1/β1 and α2/β2, respectively), whereas only the larger isoform is detected for JNKs 2 and 3.

To facilitate direct comparisons of these isoforms, we loaded one sample from ST2 cells into multiple lanes of one gel, and similarly for 3T3-L1 cells. After Western transfer, the membrane was cut and each lane was probed with an antibody to either P-JNK, JNK (total JNK), JNK1, JNK2 or JNK3. The results are shown in Figure 3D, which confirms that only the larger isoforms of JNKs 2 and 3 are present, as noted above. The JNK antibody also detects a low molecular weight band not detected by the JNK1, 2 or 3-specific antibodies. This band is unaffected by any experimental manipulations, and whether it represents a JNK or a cross-reacting protein is not known. The largest P-JNK band corresponds to the larger JNK isoform and hence could be due to phosphorylation of the larger isoforms of JNKs 1, 2 and/or 3. At the position of the smaller JNK1 isoform, the P-JNK lane shows a doublet, which has been previously described [44], [45].

Thus, although there are JNK isoform differences in the response to SRA over-expression in ST2 cells and SRA knockdown in 3T3-L1 cells, the overall pattern is one in which SRA inhibits JNK phosphorylation. Given that JNK activation (phosphorylation) inhibits insulin action in cultured cells [46], these observations support the possibility that SRA promotes adipogenesis and insulin sensitivity in part via regulation of JNK.

### Knockdown of SRA in 3T3-L1 Adipocytes is Associated with Downregulation of IR Protein and mRNA, and Decreased Downstream Insulin Signaling Pathway Phosphorylation

**Figure 4:**
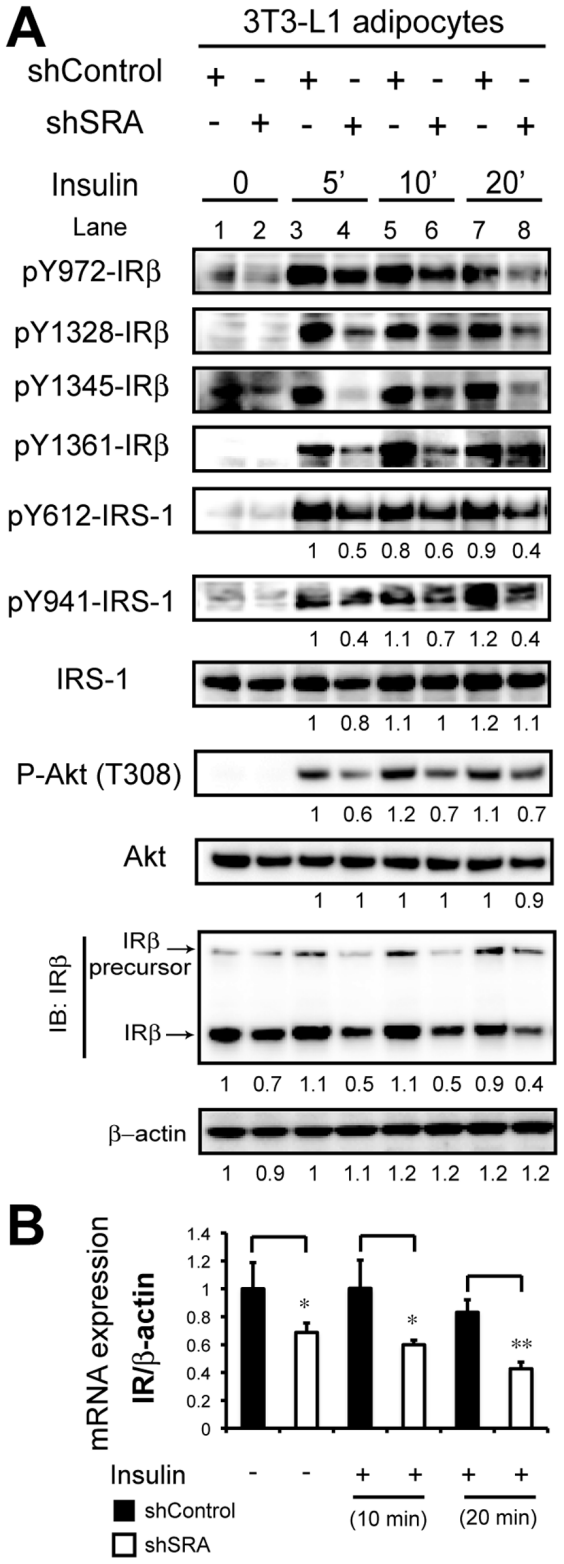
Depletion of SRA decreases autophosphorylation of insulin receptor β, tyrosine phosphorylation of IRS-1 and expression of IRβ. 3T3-L1 preadipocytes with stable knockdown of SRA (shSRA) or with shControl infection were induced to differentiate with MDIT. At day 11 post-induction, adipocytes were serum-starved for 12 h followed by treatment with or without insulin (10 nM) for the indicated durations. A, Total cell lysates were subjected to immunoblotting (IB) with antibodies against the indicated phospho-proteins or total IRβ, IRS-1, or Akt; β-actin was used as a loading control. Expression of pY612 and pY941-IRS-1, IRS-1, P-Akt, Akt, IRβ and β-actin was quantified from immunoblot digital images using Bio-Rad Quantity One software. As indicated below the IRβ blotting, expression of P-Akt and IRβ protein was normalized to expression of either Akt or β-actin and the signal of each band was compared to that for control cells as indicated (for which the level of expression was set at 1). B, Gene expression of IR mRNA at each condition was analyzed by RT-qPCR and was normalized to the expression of β-actin.

We have previously shown that the impaired insulin-stimulated glucose uptake in SRA knockdown 3T3-L1 adipocytes is associated with decreased insulin-stimulated Akt phosphorylation [29]. Here we further examine how SRA knockdown modulates insulin signaling. ShControl or shSRA knockdown 3T3-L1 preadipocytes were differentiated with MDIT and then serum-starved and treated with or without insulin. SRA/SRAP knockdown to ∼20% of control was confirmed in Figure 3B and previously [29]. The phosphorylation state of downstream targets was assessed by immunoblotting (Figure 4). We found that SRA knockdown decreased autophosphorylation of key tyrosine sites (pY972, 1328, 1345 and 1361) of IRβ, as well as downstream tyrosine phosphorylation of IRS-1 at pY612 and 941 and phosphorylation of Akt. In addition, compared to shControl adipocytes, IRβ protein content and mRNA expression in shSRA adipocytes was decreased by up to 50%, and protein expression of the IR precursor was similarly decreased (Figure 4, A & B).

**Figure 5:**
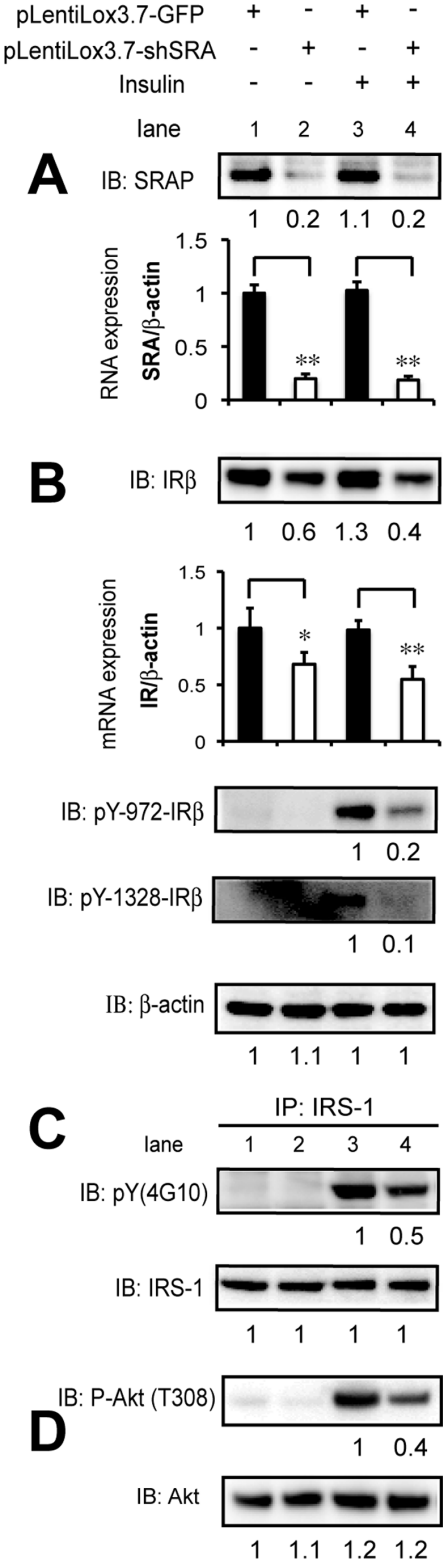
Knockdown of SRA in 3T3-L1 mature adipocytes is associated with downregulation of IR protein content and downstream insulin signaling pathway phosphorylation events. 3T3-L1 adipocytes at day 6 post-induction with MDI were infected with lentivirus expressing control (pLentiLox3.7-GFP) or SRA knockdown shRNA (pLentiLox3.7-shRNA). At day 11 post-MDI, adipocytes were starved for 12 h and treated without or with 10 nM insulin for 20 min. A, To confirm effective knockdown of endogenous SRA, an immunoblot was probed with an anti-SRAP specific antibody, and RT-qPCR was performed for SRA expression that was normalized to β-actin. B, Gene expression of IR at each condition analyzed by RT-qPCR and was normalized to the expression of β-actin. Immunoblots were subsequently performed on cell lysates from control or SRA -depleted adipocytes using antibodies as indicated. C, Similar to Figure 5A, serum-starved day 11 adipocytes were treated with or without insulin, cell lysates were first applied to immunoprecipitation for anti-IRS-1, followed by immunoblotting for anti-tyrosine (4G10). D, Immunoblots were performed for cell lysates indicated in A using Akt phospho- or total- antibodies. A–D, Quantification of each band was performed using a Bio-Rad Fluor-S MAX MultiImager. For IRβ, β-actin, IRS-1 and Akt, the signal of each band was compared to that for control cells without insulin treatment (for which the level of expression was set at 1). For tyrosine phosphorylation of IRβ and IRS-1 (pY-) and phospho-Akt, band intensity was set at 1 for insulin-treated control cells. These results are representative of three independent experiments.

We next investigated if lentiviral SRA knockdown in mature 3T3-L1 adipocytes also affects IRβ protein levels and downstream phosphorylation events. 3T3-L1 adipocytes already differentiated to day 6 post-induction were infected with lentiviruses expressing shRNA against SRA or a GFP control. SRA and SRAP effective knockdown was confirmed in Figure 5A. As found above for SRA knockdown prior to adipocyte differentiation, SRA knockdown of already-differentiated 3T3-L1 adipocytes was associated with decreased content of IRβ protein and RNA under both basal and insulin-stimulated conditions (Figure 5B, lanes 2 vs 1, and 4 vs 3). In addition, knockdown of SRA decreased insulin-stimulated tyrosine autophosphorylation of IRβ at pY972 and pY1328 (Figure 5B). Insulin-stimulated IRS-1 tyrosine phosphorylation also was reduced in SRA knockdown adipocytes, as was phosphorylation of Akt (Figure 5C and 5D).

### SRA but not SRAP Transient Expression Upregulates the IR Gene Promoter

**Figure 6:**
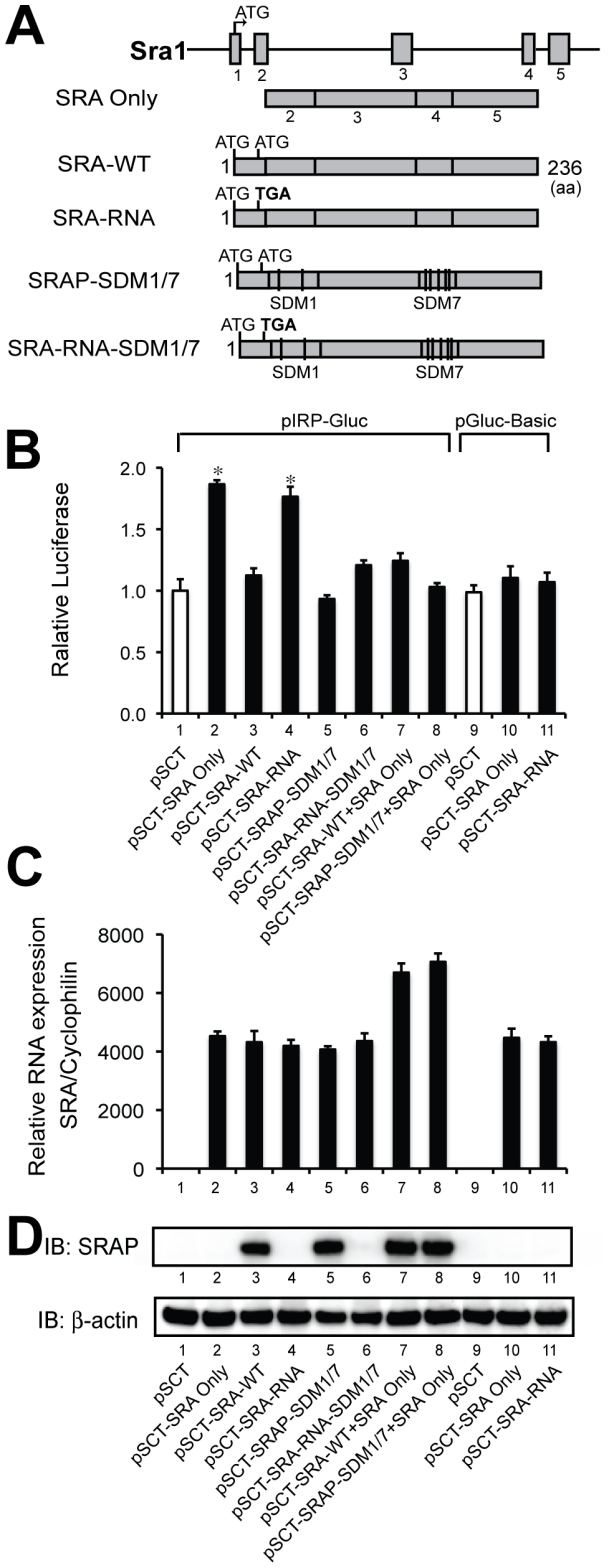
SRA not SRAP transient expression upregulates IR transcription. A, Schematic presentation of the *SRA1* gene and expression constructs used in the reporter assay. The human *SRA1* gene contains five exons as indicated by the grey boxes. “SRA Only” contains the human SRA core sequences from exons 2–5 that only express SRA RNA. SRA-WT is the full-length human Sra1 cDNA that expresses both SRAP and SRA mRNA. SRA-RNA is the SRA-WT cDNA in which codon 13 was mutated from ATG to TGA, so that SRAP is not expressed. SRAP-SDM1/7 and SRA-RNA-SDM1/7 are plasmids that are based on the SRA-WT and SRA-RNA constructs with introduction of silent nucleotide mutations in the stem loops 1 and 7, which disrupt the RNA stem loop structures (and hence impair coactivation by SRA [34]) without changing the amino acid sequence of the encoded SRAP. B, SRA upregulates IR transcription in 3T3-L1 cells. 3T3-L1 preadipocytes were cotransfected with either empty vector (pSCT, 500 ng), pSCT-SRA Only (500 ng), or other SRA/SRAP constructs (500 ng) as indicated and IR promoter (pIRP-Gluc, 100 ng in lanes 1–8) or pGluc-Basic (lanes 9–11) driven luciferase expressing plasmids. Forty-eight hours post-transfection, luciferase activity was measured. Data were expressed as fold change after normalization with the total cell protein content of each well. These results are representative of five independent experiments. Error bars indicate the standard deviation. The results of statistical analyses by ANOVA followed Scheffe’s test, comparing bar 1 with bars 2–8, and bar 9 with bars 10–11 and with bars 2 and 4, are shown in the figure (*, p<0.05). C, Confirmation of SRA overexpresion in the transient transfection in B by RT-qPCR, using human SRA primers and normalized to Ppia (cyclophilin A). The data are presented as the mean ± S.D. relative to the SRA expression in pSCT cells set at 1. D, Immunoblot using anti-SRAP indicating SRAP expression in the transient transfection in B (endogenous SRAP expression in lanes 1, 2, 4, 6, and 9–11 was visible only with a longer exposure). Immunoblotting with anti-β-actin served as a loading control.

The negative effect of SRA knockdown on IR protein content likely occurs at a transcriptional level, since SRA knockdown also decreased IR mRNA expression to a similar extent (Figure 4B & 5B). To test this hypothesis, we compared the effects of SRA RNA, SRAP and SRA/SRAP with various mutations on transcription driven by the IR gene promoter in a reporter assay. As shown in Figure 6A, these plasmids include pSCT-SRA (denoted as SRA Only), which expresses the human SRA RNA core sequence but not SRAP [24]; pSCT-hSRAP (denoted as SRA-WT), which expresses both full length SRA mRNA and SRAP; pSCT-hSRAP-RNA (denoted SRA-RNA), in which pSCT-hSRAP was mutated at codon 13 (ATG to TGA) thus preventing expression of SRAP while causing only this 3 nucleotide change in the sequence of expressed SRA RNA; and pSCT-SRAP-SDM1/7 and pSCT-SRA-RNA-SDM1/7 that contain silent mutations in SRA stem loops 1 and 7 as described previously [34]. These mutations disrupt the structures of stem loops 1 and 7, which are critical for SRA function, without altering the protein sequence of the encoded SRAP. Cotransfection of the IR promoter – luciferase reporter (pIRP-Gluc) or pGluc-Basic lacking the IR promoter sequence with SRA RNA, SRAP or the mutated constructs described above indicated that SRA Only demonstrates enhancement of pIRP-Gluc reporter activity (Figure 6B, bar 2 vs. 1), but SRA-WT that expresses both SRA mRNA and SRAP does not (bar 3). As expected, SRA-RNA (bar 4) that expresses SRA mRNA but has its 13^th^ codon mutated to a stop signal has comparable coactivation to SRA Only. SRAP-SDM1/7 that expresses a stem loop-disrupted SRA but wild type SRAP shows further decreasing activity compared to SRA only and SRA-WT (bar 5 vs. 2 and 3). In addition, SRA-RNA-SDM1/7 no longer activates transcription (bar 6 vs. 4). In addition, neither SRA Only nor SRA-RNA coactivates the reporter lacking the IR promoter sequence (bars 10 & 11 vs. 9), indicating SRA’s activity is specific for the IR promoter. In agreement with previous findings that SRAP may inhibit its own RNA function [47], our data support that SRAP may inhibit its own mRNA’s coactivation (bar 3 vs. 4). This conclusion was further substantiated in that SRA-WT (expressing both full-length SRAP and SRA mRNA) and SRAP-SDM1/7 (expressing SRAP with impaired RNA function) inhibit the coactivation of SRA Only (bars 7 & 8 vs. 2 & 4). Appropriate expression of the various SRA RNA and SRAP constructs was confirmed in Figures 6 C and D.

## Discussion

SRA has been characterized as a long non-coding RNA that enhances the transcriptional activity of steroid receptors [24], non-steroid nuclear receptors and other transcription factors [25], [26], [27], [28]. By alternative splicing, the *SRA1* gene also encodes an SRAP protein [31], [33]. SRA RNA appears to have diverse biological functions, such as in mammary gland development [48] and muscle differentiation [25], although the lack of a knockout mouse has impeded analysis of the function of SRA *in vivo*. The function of SRAP has been even more difficult to discern, in part because SRAP expression requires expression of the RNA, and in part because SRA and SRAP have been shown to exist together in a ribonucleoprotein complex [47].

We previously showed that SRA promotes adipogenesis and stimulates insulin-stimulated glucose uptake and Akt activation in adipocytes [29]. However, the mechanisms underlying these effects of SRA are not well understood. In the present study we found that SRA overexpression in ST2 adipocyte precursors significantly promotes adipogenesis, especially upon induction with hormonal cocktails that include insulin (Figure 1). This led us to investigate potential effects of SRA on insulin signaling. A combination of overexpression and knockdown studies indicate that SRA but not SRAP induces gene expression driven by the IR promoter. This results in elevated IR mRNA and protein expression, with consequent increases in insulin-stimulated phosphorylation of IRS-1 and Akt (Figure 4–5). Expression of the IR gene is regulated by numerous transcription factors, including C/EBPα/β, glucocorticoid receptor, Sp1, NF-1 and others. We speculate that SRA coactivates one or more of these factors to induce IR expression. In fact, SRA is known to coactivate the glucocorticoid receptor [24], although it has been reported not to coactivate Sp1 [24] or C/EBPα [29].

Recent studies suggest that active p38 MAPK inhibits adipogenesis [38]. Therefore, our observation of decreased p38 MAPK activation (Figure 3A) could explain the enhanced adipogenesis in SRA-overexpressing ST2 precursors. However, stable knockdown of SRA did not affect p38 activation in 3T3-L1 adipocytes (Figure 3C), even though adipogenesis is impaired in these cells [29]. Therefore, the contribution of altered p38 activity to the adipogenic effects of SRA may depend on the cell type or other factors.

In addition, we demonstrate that SRA overexpression markedly inhibits, while depletion increases, phosphorylation of certain JNK species (Figure 3). Since the absence of JNK1 enhances insulin receptor signaling [40], the inhibition of JNK activity by SRA may further activate insulin signaling and promote adipocyte differentiation. By alternative processing of RNA transcripts, the genes encoding JNK1, JNK2 and JNK3 produce 10 protein isoforms of approximately 46 and 54 kDa [42]. We noted that SRA overexpression in ST2 cells only inhibits the phosphorylation of p46 JNK (which represents JNK1), while SRA knockdown in 3T3-L1 preadipocytes primarily upregulates phosphorylation of p54 JNK (which may represent JNKs 1, 2 and/or 3) (Figure 3A, C). There are several potential explanations for these differences. The shSRA depletes both SRA (RNA) and SRAP (protein), whereas the SRA over-expression vector does not produce SRAP. The function of SRAP is unknown, but it could potentially play a role in the regulation of JNK phosphorylation. Another possibility is that p46 JNK phosphorylation may already be maximally induced by MDI in 3T3-L1 cells, so that loss of SRA cannot induce it further. Similarly, ST2 cells may contain enough endogenous SRA to maximally inhibit p54 JNK phosphorylation such that over-expression has no further effect, whereas the inhibition of p46 JNK phosphorylation may require a higher level of SRA. Alternatively, there may be cell-specific differences in the expression or availability of upstream MAPK kinases and associated proteins that are recruited to the SRA ribonucleoprotein complex.

SRA is highly expressed in breast and prostate cancer cells [31], [49]. Thus, the ability of SRA to induce IR protein expression and increase downstream phosphorylations of IRS-1 and Akt in response to insulin may occur in these and possibly other cancers. In these settings SRA/SRAP could contribute to Akt-stimulated cancer cell growth.

In summary, the present study reveals that SRA plays an intrinsic role in the regulation of adipocyte differentiation and insulin signaling, at least in part by inhibiting phosphorylation of JNK and p38 MAPK, increasing IR transcription, maintaining IRβ protein levels and enhancing downstream signaling pathways through IRS-1 and Akt.
